# Bronchial eosinophils, neutrophils, and CD8 + T cells influence asthma control and lung function in schoolchildren and adolescents with severe treatment-resistant asthma

**DOI:** 10.1186/s12931-022-02259-4

**Published:** 2022-12-09

**Authors:** Miriam Cardoso Neves Eller, Karina Pierantozzi Vergani, Beatriz Mangueira Saraiva-Romanholo, Natália de Souza Xavier Costa, Jôse Mára de Brito, Leila Antonangelo, Caroline Silvério Faria, Joaquim Carlos Rodrigues, Thais Mauad

**Affiliations:** 1 Unidade de Pneumologia Pediátrica, Instituto da Criança, Hospital das Clínicas HCFMUSP, Faculdade de Medicina, Universidade de São Paulo, Av. Dr. Eneias de Carvalho Aguiar, 647, São Paulo, SP CEP 05403-000 Brazil; 2 Universidade da Cidade de São Paulo (UNICID), São Paulo, SP Brazil; 3 Departamento de Patologia, Faculdade de Medicina, Universidade de São Paulo, São Paulo, SP Brazil; 4 Divisao de Patologia Clinica, Departamento de Patologia, Faculdade de Medicina, Hospital das Clinicas HCFMUSP, Universidade de São Paulo, São Paulo, SP Brazil; 5 Laboratorio de Investigacao Clinica (LIM03), Faculdade de Medicina, Universidade de São Paulo, São Paulo, SP Brazil; 6 Departamento de Patologia E Parasitologia, Universidade Federal de Alfenas, Alfenas, MG Brazil

**Keywords:** Severe asthma, Children, Inflammatory cells profile, Sputum, Endobronchial biopsy, CD8 + T cell

## Abstract

**Background:**

Studies in adult severe treatment-resistant asthma (STRA) have demonstrated heterogeneous pathophysiology. Studies in the pediatric age group are still scarce, and few include bronchial tissue analysis.

**Objective:**

We investigated 6–18-year-old patients diagnosed with STRA in Sao Paulo, Brazil, by characterizing the different lung compartments and their correlations with asthma control and lung function.

**Methods:**

Inflammatory profiles of 13 patients with a confirmed diagnosis of STRA were analyzed using blood, induced sputum, bronchoalveolar lavage, viral and bacterial screens and endobronchial biopsy. Inflammatory cells, cytokines, and basement membrane thickening were tested for correlations with the asthma control test (ACT) and spirometry and plethysmography parameters.

**Results:**

Endobronchial biopsy specimens from 11 patients were viable for analysis. All biopsies showed eosinophilic infiltration. Submucosal (SM) eosinophils and neutrophils were correlated with worse lung function (pre-BD FEV1), and SM neutrophils were correlated with fixed obstruction (post-BD FEV1). Intraepithelial (IE) neutrophils were positively correlated with lung function (pre-BD sGaw). CD8 + T cells had the highest density in the IE and SM layers and were positively correlated with ACT and negatively correlated with the cytokines IL1β, IL2, IL5, IL7, IL10, IL12, IL17, GCSF, MCP-1, INF-δ, and TNFα in sputum supernatant. The ASM chymase + mast cell density correlated positively with quality-of-life score (pAQLQ) and ACT.

**Conclusion:**

Eosinophils and SM neutrophils correlated with worse lung function, while IE neutrophils correlated with better lung function. Most importantly, CD8 + T cells were abundant in bronchial biopsies of STRA patients and showed protective associations, as did chymase + mast cells.

**Supplementary Information:**

The online version contains supplementary material available at 10.1186/s12931-022-02259-4.

## Introduction

Asthma is a chronic inflammatory disease of the airways, with multiple clinical presentations (phenotypes) involving different inflammatory mechanisms (endotypes) [1]. Children and adolescents with severe asthma are predominantly atopic, with high levels of total IgE and sensitization to aeroallergens [1]. However, the inflammatory phenotypes, degree of inflammation, and changes in lung function can vary considerably from patient to patient [1].

It is estimated that even after optimization of maintenance anti-inflammatory treatment and monitoring of adherence, approximately 2.5% of children with asthma will have severe treatment-resistant asthma (STRA) [2]; moreover, this population has high morbidity and health-care costs [3]. Due to the pathophysiological heterogeneity of STRA, it is thought to involve different cell types and inflammatory pathways, so a single therapeutic strategy might not be successful for all individuals [4].

Viral and bacterial infections can influence the development of asthma in children, especially in the first years of life, and are related to exacerbations at any age. Among viruses detected, human rhinovirus (RV), respiratory syncytial virus (RSV), enteroviruses, human bocavirus, influenza viruses, human parainfluenza viruses, and coronaviruses have all been associated with asthma exacerbations in humans [5]. Bacterial species that have been linked with asthma exacerbations include *Haemophilus influenzae*, *Streptococcus pneumoniae*, *Moraxella catarrhalis*, *Mycoplasma pneumoniae*, and *Chlamydia pneumoniae* [5]. Eosinophils can capture and inactivate viruses, but patients with severe asthma seem to have reduced antiviral function of eosinophils, contributing to higher viral loads [6].

Eosinophilic atopic asthma has always been considered to be mediated by the Th2 response, wherein CD4 + T cells play a major role in the production of the Th2 cytokines IL4, IL5, and IL13. However, in children with severe eosinophilic asthma, Bossley et al. demonstrated an absence of Th2 mediators in bronchoalveolar lavage (BAL) fluid, induced sputum, and endobronchial biopsies [4, 7]. Studies applying in-depth molecular techniques and immune profiling to the airways or BAL fluid have shown a more complex disease with distinct but also overlapping endotypes, including Th1 and Th17 [5].

The role of neutrophils in pediatric STRA is still not completely understood. In adult patients with severe asthma, neutrophils are related to poor response to corticosteroid therapy [8]. Additionally, airway neutrophilia has been associated with asthma exacerbation, reduced airflow, and death [9]. Nevertheless, the role of neutrophils in children and adolescents with STRA remains controversial, as intraepithelial neutrophils correlate with better lung function [10].

Inflammation in asthma may be assessed by invasive and noninvasive techniques, as each method samples different compartments of the lungs or the periphery [4]. In adults, the analysis of endobronchial biopsies has greatly advanced our understanding of the inflammatory phenomena in severe asthma [11], but few studies have phenotyped the mucosal inflammation of pediatric populations with STRA. Sputum, BAL and biopsies represent different lung compartments, and their results might not always correlate with each other. Bronchial biopsies reflect submucosal inflammation, whereas BAL and sputum represent the airway lumen only. Further, the analysis of bronchial biopsies provides information on the structural changes of the airways, which also influence asthma characteristics.

Latin American cities face several socioenvironmental inequities, with a high prevalence and severity of asthma [12]. However, there are no data regarding systemic and lung inflammation in children and adolescents with therapy-resistant asthma and its clinical repercussions in Latin American cities. Therefore, we investigated 6–18-year-old patients diagnosed with STRA in São Paulo, Brazil, by characterizing their blood, sputum, bronchoalveolar lavage, and mucosal inflammatory profile and their correlations with asthma control and lung function. We hypothesized that endobronchial biopsies would be the airway samples best correlated with parameters of asthma control and lung function in STRA patients.

## Methods

Children and adolescents (aged 6–18 years) with severe asthma (according to the Global Initiative for Asthma criteria [13]) who were followed up for at least 6 months in our outpatient center in Sao Paulo were recruited as previously described [14] as a convenience sample. None of the patients participated concomitantly in clinical trials on asthma medications. Children previously diagnosed with cystic fibrosis, alpha-1 antitrypsin deficiency, primary ciliary dyskinesia, obliterative bronchiolitis, or bronchopulmonary dysplasia were not enrolled. Patients with any significant nonpulmonary diseases or who could not adequately perform pulmonary function tests were also excluded. All patients and their parents were informed about the objectives and possible risks involved, and the parents of each child signed an informed consent form. The study was approved by the Ethics Committee for Analysis of Research Projects, Hospital das Clínicas, School of Medicine (USP no. 0650/10).

All patients were assessed by clinical interviews, physical examination, the asthma control test (ACT), and the Pediatric Asthma Quality of Life Questionnaire (pAQLQ) [15] and were monitored for adherence to treatment at three consecutive visits (Additional file 1: Figure S1) [14]. After the third follow-up visit, the patients were assessed for asthma control according to the European Respiratory Society/American Thoracic Society (ERS/ATS) criteria [16]. The participants were confirmed to have asthma with poor symptom control [17, 18] despite receiving high doses of inhaled corticosteroids (≥ 800 µg day budesonide equivalent) and optimization of underlying modifiable factors, such as adherence [16]. Children underwent bronchoscopy for research purposes with BAL collection and endobronchial biopsy sampling. Bronchoscopy was performed under general anesthesia with sevoflurane, propofol, fentanyl and lidocaine topically. After the procedure, patients were re-evaluated and treated as needed. Materials were processed in both clinical (bacterial culture and viral PCR in BAL fluid) and research laboratories (sputum analyses, cell and structural quantifications in bronchial biopsies and cytokine analyses).

### Pulmonary function tests

Pre- and postbronchodilator (400 mcg salbutamol) spirometry and plethysmography were performed using a SensorMedics Vmax® (VIASYS Healthcare; Yorba Linda, CA) device on the first and third visits using techniques standardized by the ERS/ATS [19]. Global Lung Function Initiative [20] reference values were used for spirometry, and Polgar and Weng’s reference values were used for plethysmography [21].

### Measurement of fractional exhaled nitric oxide

Online measurement of fractional exhaled nitric oxide (FeNO) was performed by measuring the chemiluminescence of a single blow at the expiratory flow of 50 ml/s using a NIOX MINO® device prior to plethysmography and sputum collection. The ATS methodological guidelines and FeNO level classification were used [22].

### Sputum induction

Absence of asthma exacerbation was confirmed before induction. Patients with FEV1 ≥ 65% of predicted were subjected to four nebulizations with hypertonic (3%) saline solution [23]. For stable patients with FEV1 < 65% of the predicted value, nebulization was performed using normal saline (0.9% NaCl). Sputum processing was performed as described by Pizzichini et al. [24] except that the supernatant for cytokine analysis was obtained without the addition of 0.1% dithiothreitol, which was added later for cellular analysis. Cell viability and total cell count were analyzed using a Neubauer chamber under an optical microscope; only samples with nonviable and squamous cell percentages less than 50–80% were considered [25].

### Cytokine level measurements

Cytokines were quantified in BAL fluid, sputum supernatants and plasma by using the Luminex multiplex bead analysis system. The premade Bio-Plex Pro™ Human Cytokine 17-plex immunoassay analysis system (Bio-Rad Laboratories, Hercules, CA, USA) was used to detect interleukin (IL)-1, IL-2, IL-4, IL-5, IL-6, IL-7, IL-8, IL-10, IL-12, IL-13, IL-17, granulocyte colony-stimulating factor (GCSF), granulocyte–macrophage colony-stimulating factor (GM-CSF), interferon (IFN)-γ, monocyte chemoattractant protein (MCP)-1, macrophage inflammatory protein (MIP)-1β, and tumor necrosis factor (TNF)-α [26]. The samples were analyzed in a Bio-Plex 200 device (Bio-Rad Laboratories). The concentrations of cytokines were estimated from a standard curve using Bio-Plex Manager™ Software (Bio-Rad Laboratories).

The minimum levels of detection of the different cytokines were IL-1: 13.7 pg/mL; IL-2: 1.24 pg/mL; IL-4: 0.45 pg/mL; IL-5: 2.18 pg/mL; IL-6: 1.38 pg/mL; IL-7: 1 pg/mL; IL-8: 1.23 pg/mL; IL-10: 0.71 pg/mL; IL12: 5.75 pg/mL; IL-13: 1.14 pg/mL; IL17: 2.9 pg/mL; G-CSF: 6.76 pg/mL; GM-CSF: 4.12 pg/mL; IFN-γ: 1.31 pg/mL; MCP-1: 2.35 pg/mL; MIP-1β: 2.9 pg/mL; TNF-α: 3.11 pg/mL.

### Bronchoscopy, BAL, and endobronchial biopsy

Examinations were performed on an outpatient basis at the *Instituto da Criança/HCFMUSP*. The patients had to be stable and free of exacerbations for at least two weeks without the use of systemic corticosteroids. Bronchoscopy was performed on STRA patients using a flexible bronchoscope (diameter, 5 mm; working channel, 2 mm) under general anesthesia. BAL was collected from the middle lobe using 3 aliquots of up to 1 mL/kg body mass of saline. The first sample was used for microbiological analysis. Endobronchial biopsy was obtained from the secondary carina of the right upper lobe. An average of six samples were collected per patient, which were and processed as follows: (1) stored in RNAlater solution (Sigma-Aldrich) at − 80 °C (n = 2), (2) formalin fixed and paraffin-embedded (n = 2), or (3) glutaraldehyde fixed for electron microscopy (n = 2). Patients were monitored after the procedure until complete recovery and were discharged after careful reassessment by the anesthetist and attending physician. All procedures were supervised by a physician who did not participate in the study to ensure that the examination could be interrupted, if necessary, to avoid any undue risk to the patient. There were no observed anatomic abnormalities or serious adverse effects; one patient had laryngospasm after the procedure, which resolved quickly.

### BAL analyses

Slides were prepared using cytocentrifuged BAL samples, and Giemsa staining was used for differential cell counting. Cell viability was analyzed using trypan blue; cell counts were obtained using a Neubauer chamber, and the results are expressed as cells/mm^3^ [27]. BAL samples were stored at − 70 °C and then analyzed for 12 respiratory viruses, *Mycoplasma pneumoniae*, *Chlamydophila pneumoniae*, and *Bordetella pertussis,* using the multiplex real-time PCR assay FilmArray® Respiratory Panel (BioFire, Utah, US). FilmArray extracts and purifies all nucleic acids from the sample and performs nested multiplex PCR. Individual singleplex second-stage PCRs detect the products from the first-stage PCR. The method has an overall sensitivity and specificity of 95% and 99%, respectively [28].

### Bronchial tissue processing and immunohistochemistry

Biopsy samples were fixed in 4% buffered formalin for 24 h, embedded in paraffin, sectioned into 3-μm-thick sections, and then stained with hematoxylin & eosin and Alcian Blue-periodic acid of Schiff for the assessment of basement membrane (BM) thickness and Congo red for eosinophil counts. The immunohistochemistry parameters are shown in Additional file 1: Table S1. Briefly, the sections were dewaxed and rehydrated, and antigen retrieval was carried out as described. Prior to overnight incubation with the primary antibody, the sections were incubated in 3% H_2_O_2_ for 30 min to inhibit endogenous peroxidase activity. Then the sections were incubated with Polymer Novolink (Leica Biosystems Newcastle, UK) secondary antibody at 37 °C for 30 min. Diaminobenzidine was used as a chromogen (Sigma-Aldrich Chemie, Steinheim, Germany). All slides were counterstained with Harris hematoxylin (Merck, Darmstadt, Germany) [29].

### Biopsy analysis

Two biopsies per patient were analyzed. Biopsies that were too exiguous and those with areas of artifactual hemorrhage or crushed tissue were not included in the study. The tissue samples were analyzed in a blinded manner, and all slides were digitized using a 3DHistech Slide Scanner (3DHistech, Budapest, Hungary). Eosinophils, neutrophils, tryptase-positive and chymase-positive mast cells, and CD4 + and CD8 + T lymphocytes in the submucosal (SM), airway smooth muscle (ASM), and intraepithelial layers were quantified using Case Viewer for Windows (3DHistech, Budapest, Hungary); for intraepithelial analyses, cells were quantified in completely intact or semi-intact epithelium present in each analyzed airway. The SM layer was defined as that located between the epithelium and the internal ASM border, including the BM and lamina propria [30]. All analyses were performed as previously described by Ferreira et al., 2018 [29]. BM thickness and length were measured using Image-Pro® Plus 4.1 (Media Cybernetics, Silver Spring, MD, USA).

### Statistical analysis

The sample was opportunistic because no previous data were available to inform a power assessment. The distribution of continuous variables was assessed using the Kolmogorov‐Smirnov test. Descriptive data are presented as the mean (SD) or median (interquartile range), depending on the data distribution. Correlations were assessed using Spearman's correlation coefficient. P‐values < 0.05 were considered statistically significant. Data visualization and statistical analysis were performed using SPSS 21.0 (SPSS, Chicago, IL, USA). Correlation plots were created using RStudio, version 1.4.1106 (RStudio, PBC, Boston, MA, USA).

## Results

Forty-five patients with severe asthma were recruited for this study. At the end of the protocol, thirteen patients (nine males, four females; median age 12 years, range 9–17 years) were diagnosed with STRA according to the ERS/ATS criteria [16] and underwent bronchoscopy. The demographic, clinical, and laboratory characteristics and treatment data of these thirteen subjects are presented in Table 1.

**Table1:** Characteristics of patients with severe therapy-resistant asthma (STRA) n = 13

Variables	STRA N = 13
*Clinical and demographic data at visit 1*	
Age in years, mean (SD)	12.45 (2.73)
Male sex (%)	69.3%
Age at onset of symptoms in months, mean (SD)	12.5 (2.7)
Mean BMI* Z score (SD)	0.9369 (1.092)
Mean height Z score (DP)	-0.200 (0.9615)
Exposure to smokers (%)	23%
Almost fatal crisis (%)	38.4%
Obesity (%)	11.2%
Sinusopathy (%)	42.3%
*Treatment*	
Budesonide 800 mcg/day (%)	92.3%
Fluticasone 500 mcg/day (%)	7.7%
Oral steroid (Visit 1) (%)	7.8%
LABA (%)	100%
LTRA (%)	69.2%
Nasal steroid (%)	100%
Good adherence (≥ 85%) to treatment- visit 1 (%)	69.2%
Good adherence (≥ 85%) to treatment -visit 3 (%)	84.6%
*Follow-up data (visit 3)*	
ACT ** – mean (SD) visit 1	15,3 (3,43)
ACT ** – mean (SD) visit 3	15,5 (3,77)
Atopy *** (%)	92%
Serum IgE IU/ml median (min–max)	811.0 (4.0–4.740.0)
*Pulmonary function tests – mean (SD) (visit 3)*	
Pre-BD FEV1	82.87% (24.35)
Pre-BD FVC	99.74 (19.48)
Pre-BD FEF 25–75%	57.73% (30.03)
Pre-BD FEV1/SVC	72.15% (13.26)
Pre-BD TLC	98.50% (13.24)
Pre-BD RV	130.4% (56.79)
Pre-BD RV/TLC	23.92% (11.66)
Pre-BD Resistance	150.5% (74.34)
Pre-BD Conductance	44.75% (20.26)
Post-BD variation in FEV1 – median (IQR)	14% (6,75%–19%)
Post-BD variation in conductance – median (IQR)	60,2% (36,2%-93,9%)
*Noninvasive assessment of inflammation*	
Blood eosinophils/mm^3^ median (min–max)	533.9 (96.2–2.202.9)
FeNO (ppb) median (min–max)	31.0 (7.0–103.0)
Sputum total cells × 10^6^ (visit 1) median (min–max)	1.86 (0.3–5.0)
Sputum total cells × 10^6^ (visit 3) median (min–max)	3.60 (1.2–6.6)
% Sputum Eosinophils (visit 1) median (min–max)	14,1% (0,4–65,2)
% Sputum Eosinophils (visit 3) median (min–max)	3,48% (0–66,67)
% Sputum neutrophils (visit 1) median (min–max)	43,6% (4,2–87,6)
% Sputum neutrophils (visit 3) median (min–max)	44,68% (7,6–74,5)

*BMI: body mass index; **ACT: asthma control test; ***Atopy: at least one positive allergen in the skin prick test. Functional pulmonary data are expressed as the mean percentages of predictions and standard deviations according to the Global Lung Function Initiative reference values [12] for spirometry and Polgar and Weng’s reference values for plethysmography [13]. LABA: long-acting β2 agonist; LTRA: leukotriene receptor antagonist; BD: bronchodilator; FEV1: forced expiratory volume in the first second; FVC: forced vital capacity; FEV1/SVC: ratio of forced expiratory volume in the first second to slow vital capacity; FEF 25–75%: forced expiratory flow at 25–75% of FVC; TLC: total lung capacity; RV: residual volume; RV/TLC: ratio of residual volume to total lung capacity; IgE: immunoglobulin E; FeNO: fractional exhaled nitric oxide measured at 50 L/min

**Fig. 1:**
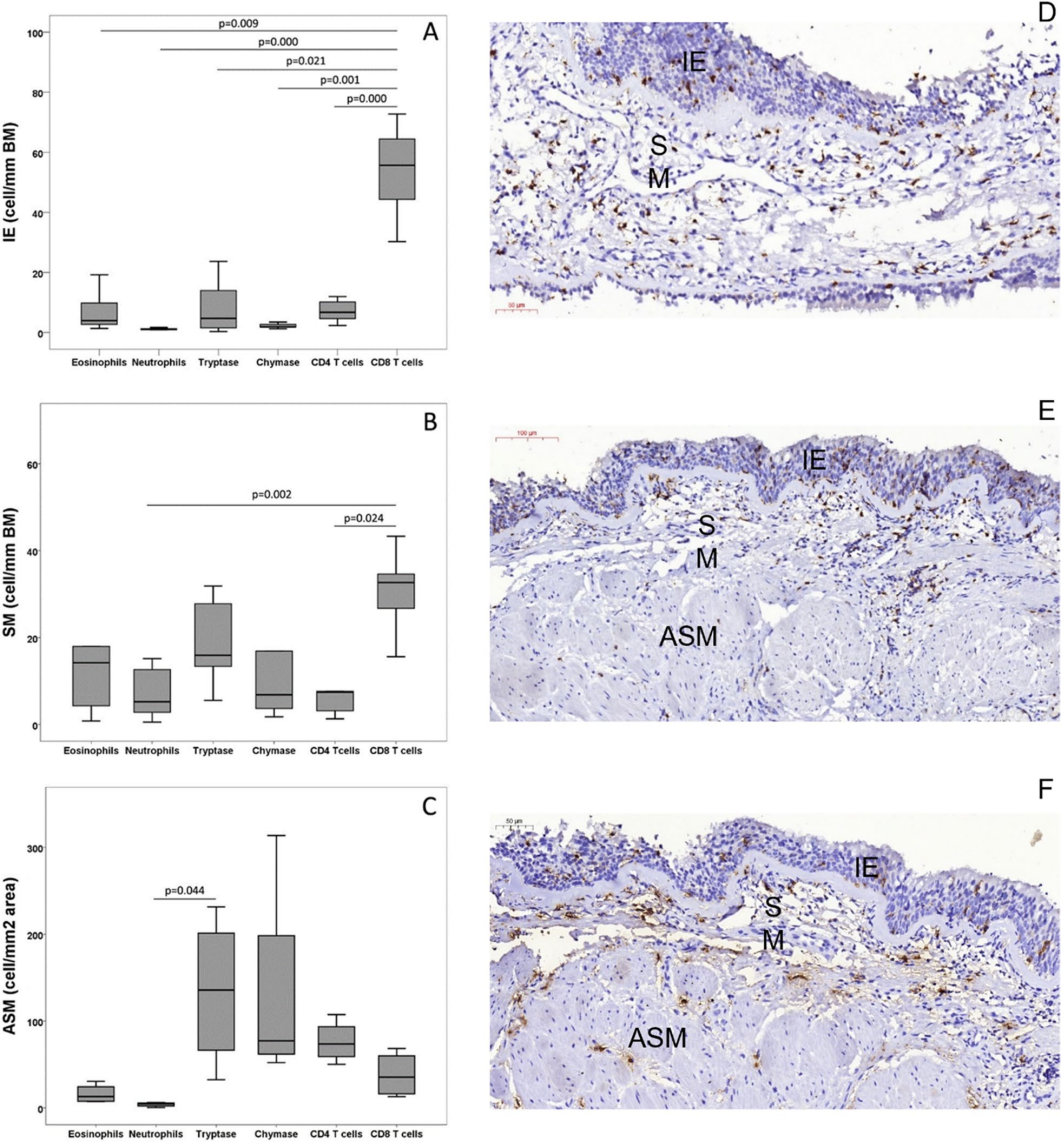
The graphs (**A**–**C**) show different inflammatory cell densities by compartment in bronchial biopsies of patients with severe therapy-resistant asthma (STRA) (n = 11). **A** IE. intraepithelial; **B**: SM. submucosal; **C**: ASM. airway smooth muscle. As examples, the figures show CD8 + T cells (**D**, **E**) and tryptase + mast cells (**F**) in different compartments of the airways

Endobronchial biopsy specimens from 11 patients were viable for analysis, whereas two had exiguous tissue insufficient for analysis. Histological analysis revealed that the biopsies were mostly representative of the epithelial and submucosal layers, although eight biopsies were representative of ASM. The epithelial layer was partially detached, with patchy areas of goblet cell hyperplasia. Submucosal inflammation varied from mild to moderate, and most biopsies showed eosinophilic infiltration. Descriptive analyses of cell counts are presented in Table 2. In the intraepithelial layer, CD8 + T-cell density was higher than those of all other quantified cell types (p = 0.021); CD8 + T-cell density was also highest in the SM layer, with statistically significant differences compared to that of neutrophils (p = 0.002) and compared to the density of CD4 + T cells (p = 0.020). In the ASM layer, the tryptase + mast cell density was the highest, with a statistically significant difference compared to that of neutrophils (p = 0.004; Table 2, Fig. 1).

**Table 2:** Cell density per layer (intraepithelial: IE, submucosal: SM, and airway smooth muscle: ASM) in bronchial biopsies of patients with severe therapy-resistant asthma (STRA) (n = 6 to 11)

Variables	N	Median	Interquartile range
			25–75%
Tissue area (mm^2^)	11	1.62	1.24**–**2.39
Thickness MB (µm)	10	7.66	6.74**–**8.30
Length (µm)	10	10,217	8535**–**14,664
IE (cell/mm BM)			
Eosinophils	11	3.94	1.38**–**14.03
Neutrophils	9	1.24	0.98**–**1.66
Tryptase	9	4.71	0.66**–**20.23
Chymase	9	1.85	1.70**–**3.49
CD4 T cells	9	6.73	2.87**–**10.29
CD8 T cells	8	55.91	44.34**–**72.32*
SM (cell/mm BM)			
Eosinophils	11	9.68	2.63**–**18.00
Neutrophils	11	4.46	1.27**–**12.65
Tryptase	10	17.99	13.38**–**27.81
Chymase	10	9.72	3.67**–**16.88
CD4 T cells	10	7.03	1.64**–**7.65
CD8 T cells	10	31.56	24.04**–**34.60**
ASM (cell/mm^2^ area)			
Eosinophils	6	7.92	7.13**–**18.05
Neutrophils	7	4.03	0.21**–**6.01
Tryptase	8	135.74	65.56**–**230.53^**#**^
Chymase	7	71.45	52.20**–**103.32
CD4 T cells	6	59.01	5.98**–**79.43
CD8 T cells	7	51.47	19.32**–**133.39

IE: Intraepithelial; SM: Submucosal; ASM: Airway smooth muscle; BM: Basement membrane. IE [*CD8 ≠ neutrophils (p = 0.001), tryptase (p = 0.009), chymase (p = 0.021), eosinophils and CD4 + (p = 0.000)]; SM [**tryptase ≠ neutrophils (p = 0.004); ASM [^**#**^CD8 +  ≠ neutrophils (p = 0.002) and CD4 + (p = 0.020)]

Unfortunately, the BAL samples showed higher than acceptable numbers of epithelial cells and high bacterial contamination, possibly from the oropharynx, which compromised cellular evaluation. Nine patients had positive BAL cultures (*Streptococcus pneumoniae*, n = 1; *Aspergillus fumigatus*, n = 1; polymicrobial, n = 7). Regarding RT-PCR FilmArray®-based detection of viral and atypical bacteria, only one tested positive for coronavirus NL62. The relationship between BAL and the number of cells per compartment in bronchial biopsies of patients with STRA is presented in Additional file 1: Table SII, and the significant correlations of BAL cytology and cytokines with lung function are presented in Additional file 1: Table SIII.

The positivity of bacterial cell cultures and virus and BAL granulocyte patterns is also shown in Additional file 1: Table SIV.

### Correlations between pathology and clinical, functional, and laboratory parameters:

#### Eosinophils and neutrophils

**Fig. 2:**
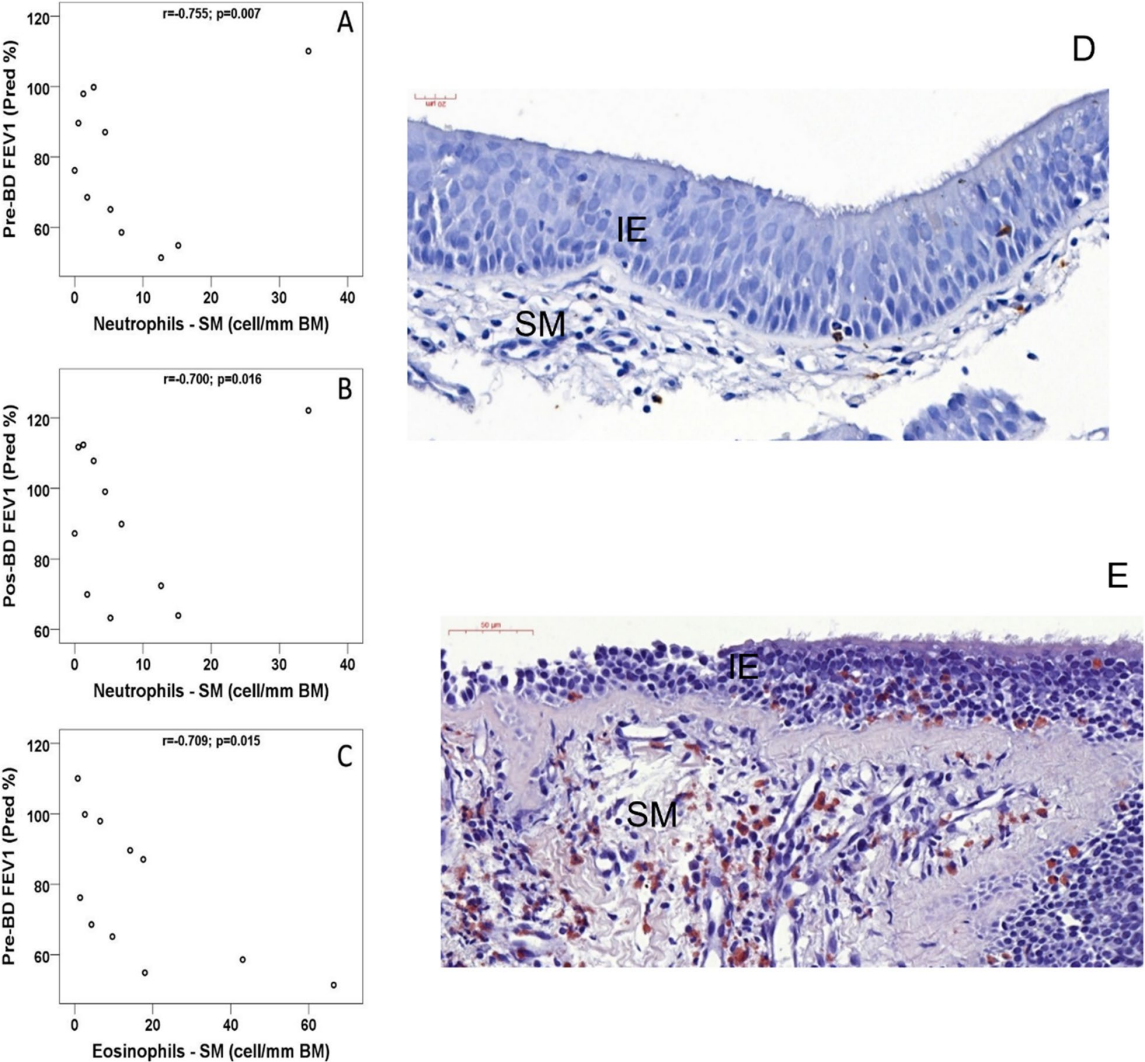
Negative correlation of submucosal neutrophils with the pre-BD FEV1 value (predicted%) (**A**) and post-BD FEV1 (**B**) and negative correlation of submucosal eosinophils with the pre-BD FEV1 value (predicted%) (**C**). BD: bronchodilator; FEV1: forced expiratory volume in the first second. Pred%: percentages of predictions according to the Global Lung Function Initiative reference values [20]. **D** and **E** Representative examples of submucosal and intraepithelial neutrophils and eosinophils (Congo Red staining) in bronchial biopsies

SM eosinophil and neutrophil densities were negatively correlated with the pre-BD FEV1 value (predicted%) (r = − 0.709, p = 0.015 and r = − 0.755; p 0.007, respectively) (Fig. 2) and the FEV1/SVC ratio (r = − 0.664, p 0.026 and r = − 0.636; p 0.035, respectively). SM neutrophil density was negatively correlated with post-BD FEV1 (r = − 0.700, p = 0.016) (Fig. 2, Table SV), but intraepithelial neutrophil density was positively correlated with pre-BD airway conductance (sGaw) (r = 0.667, p 0.005). SM eosinophil density was positively correlated with FEV1 variation post-BD (r = 0.720, p = 0.013) (Table SV). BAL neutrophil density was also inversely correlated with post-BD FEV1 (r = − 0.566, p = 0.044) (Table SIII).

### CD8 + and CD4 + cells

**Fig. 3:**
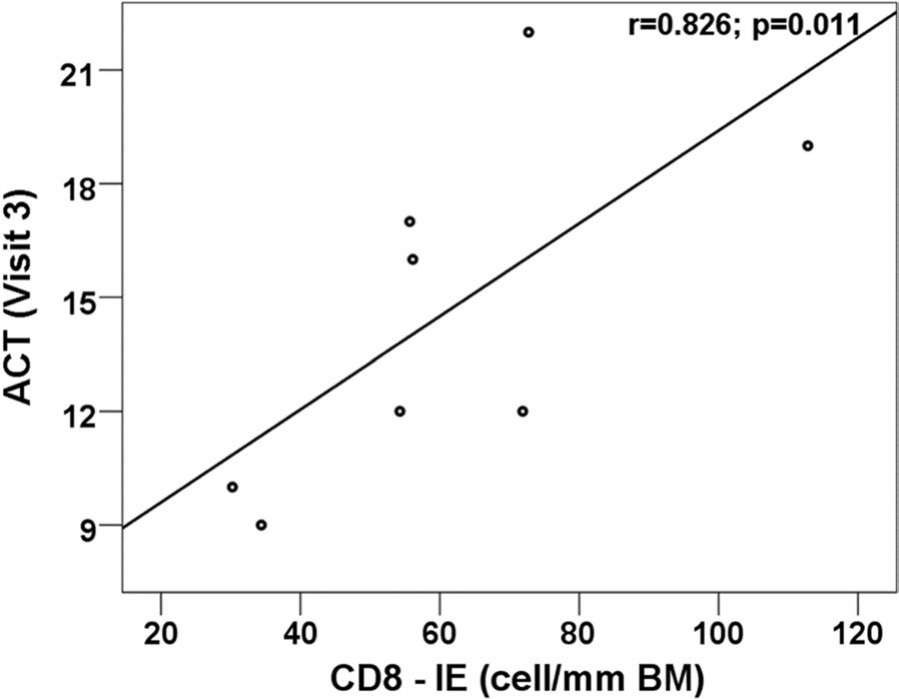
Positive correlation between CD8 + intraepithelial cells and control score (ACT) r = 0.826; p = 0.011

Intraepithelial CD8 + T-cell density was positively correlated with the symptom control score (ACT) (r = 0.829, p = 0.11) (Fig. 3), and SM CD8 + T-cell density was negatively correlated with IL1β, IL2, IL5, IL7, IL10, IL12, IL17, GCSF, MCP-1, INF-δ, and TNFα levels in the sputum supernatant (Fig. 5, Additional file 1: Figure S2). Intraepithelial CD4 + T-cell density was positively correlated with sputum IL4 (r = 0.757, p = 0.049) and GM-CSF (r = 0.786, p = 0.036) (Additional file 1: Table SVI) levels.

### Chymase and tryptase + mast cells

**Fig. 4:**
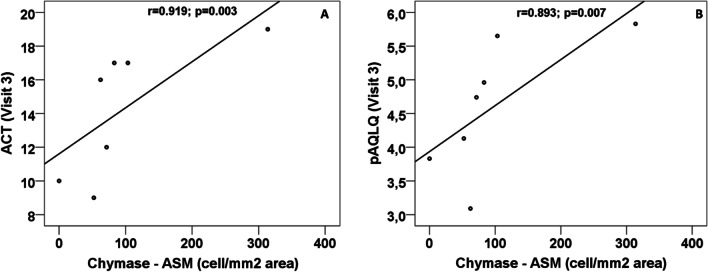
Correlation between ASM chymase (cells/mm^2^ area) and asthma control in terms of ACT (**A**) and quality of life pAQLQ (**B**)

There were positive correlations between ASM and SM tryptase + mast cell density and BM thickness (r = 0.786 p = 0.036 and r = 0.697, p = 0.025, respectively). Interestingly, chymase + mast cell density correlated positively with pAQLQ (r = 0.857, p = 0.014) and ACT (r = 0.919, p 0.003) (Fig. 4).

The median BM thickness was 7.66 µm (range, 6.74–8.30), which correlated positively with blood eosinophil density (r = 0.903, p < 0.0001) (Table SVII) and sputum IL-4 and MCP-1 levels (r = 0.894, p = 0.041 and r = 0.900, p = 0.037, respectively) (Table SVI).

**Fig. 5:**
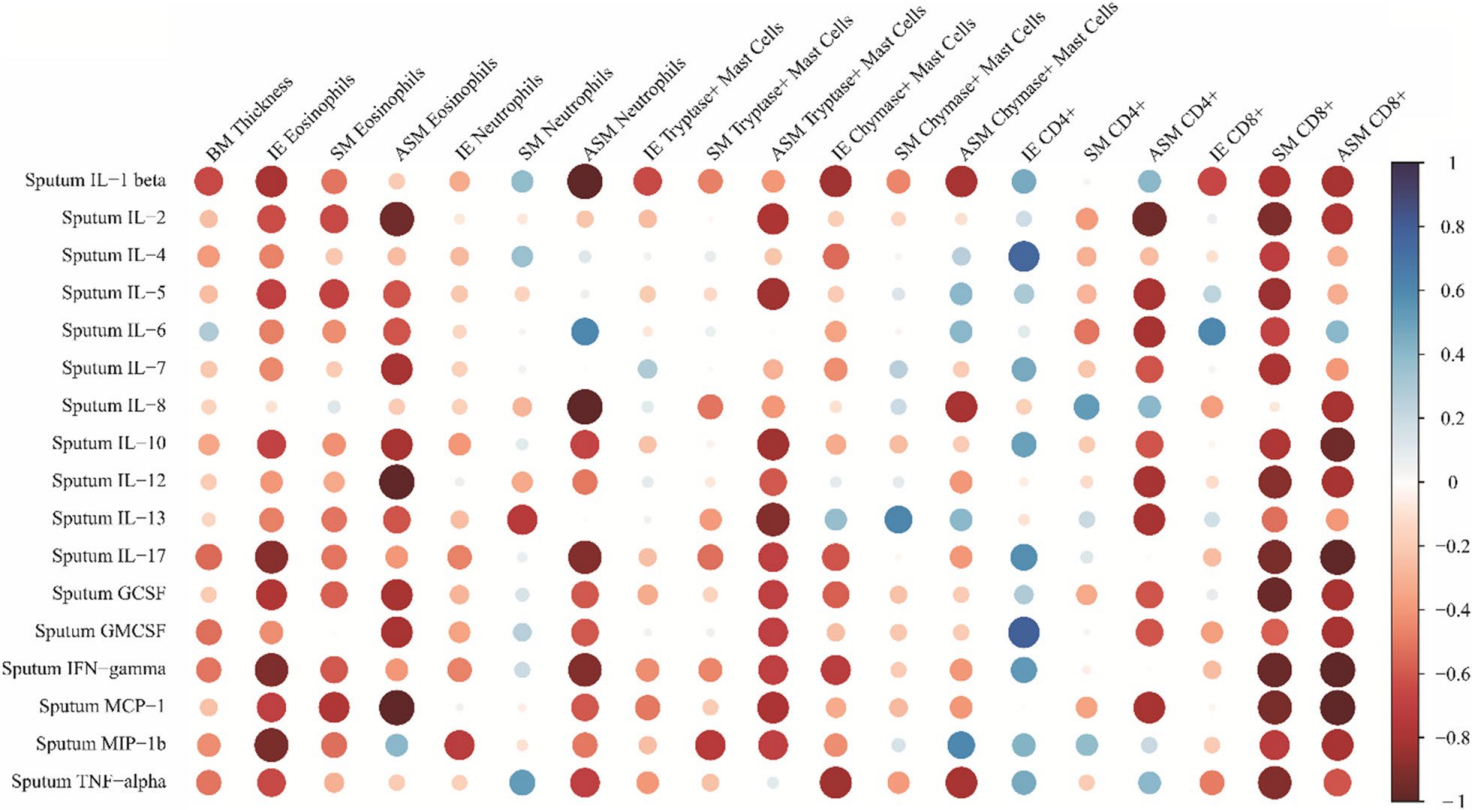
Heatmap of correlations of sputum cytokine levels with biopsy features. The correlation coefficients are color-coded from deep red (− 1) to deep blue (1)

Correlation plots for all cytokines showed only a few correlations between the three compartments BAL, sputum, and plasma (Additional file 1: Figure S3). The sputum cytokines correlated best with the biopsy cells, as seen on the heat map (Fig. 5).

## Discussion

In this study, we characterized the blood and airway inflammatory profiles of 13 schoolchildren and adolescents with STRA through sputum analysis, BAL, and endobronchial biopsies. Our data showed that they were characterized by persistent blood and airway eosinophilic inflammation, but other cell types, such as neutrophils, mast cells and CD8 + T cells, correlated with parameters of lung function and disease control. Compared to those of other compartments, bronchial tissue parameters had better correlations with functional and asthma control indicators, reinforcing the value of this tool in better understanding the pathogenesis of pediatric severe asthma [11] to improve future treatments.

Both eosinophil and neutrophil densities correlated with worse lung function; eosinophils were associated with a better bronchodilator response, and neutrophils were associated with fixed obstruction. Surprisingly, CD8 + T cells were the inflammatory cells with the highest density in the airway walls and had a protective, positive association with asthma control. Chymase + mast cells, but not tryptase + mast cells, correlated with better asthma control and quality-of-life score.

Few studies have concomitantly analyzed blood, induced sputum, BAL, and biopsies from children with severe asthma. We observed weakly significant correlations between the inflammatory cell densities or cytokine levels of different lung compartments, except for sputum cytokine levels and CD4 + and CD8 + T lymphocyte densities in biopsies. These results reinforce the potential of this minimally invasive technique for studying markers of inflammation in the different severe asthma endotypes [31].

Neutrophils are associated with asthma severity in adults [8], but their role in pediatric asthma is unclear [10]. We have previously shown that the sputum neutrophil percentage was higher in this group of children with STRA than in children with controlled severe asthma [14]. Here, we expand on those findings and demonstrate that airflow limitation was negatively correlated with submucosal neutrophil density, as observed in adults with severe asthma [32]. Conversely, the intraepithelial neutrophil count was positively correlated with sGaw. Similarly, a previous study in children with severe asthma showed that intraepithelial neutrophil density was positively correlated with lung function and better symptom control, despite lower inhaled corticosteroid doses [10]. Several neutrophil phenotypes have been described regarding their inflammatory potential [33]. In children with neutrophil-predominant severe asthma, BAL neutrophils had an impaired respiratory burst and enhanced expression of markers of activation, degranulation, and survival [34]. Intraepithelial neutrophils are probably migratory cells; moreover, aged migrating neutrophils efficiently eliminate pathogens without increased reactive oxygen species or cytokine production [35]. It is possible that this phenotypic heterogeneity accounted for the observed differences between neutrophil airway microlocation and lung function in our study.

Eosinophil density in bronchial tissue, but not in other compartments, was correlated with worse lung function in our study, which is not consistent with other studies on children with STRA [7]. This discrepancy might be explained by the different corticosteroid regimens; we chose not to administer systemic corticosteroids before the biopsy. In contrast, tissue eosinophil density correlated with a better response to bronchodilators. A longitudinal and prospective National Heart, Lung, and Blood Institute–funded Severe Asthma Research Program study showed that blood eosinophil number predicted severe asthma resolution within 3 years [36]. Blood eosinophil density was correlated with BM thickness, reinforcing the role of blood eosinophils as a biomarker of asthma, including asthma-related remodeling.

Notably, CD8 + T-cell density in the submucosa and intraepithelial region was negatively correlated with the sputum levels of many cytokines and positively associated with a better ACT. To our knowledge, this is the first time that such associations have been reported in severe pediatric asthma in children who did not have a viral infection. The children enrolled in this study lived in São Paulo, a city that constantly reports high air pollution levels [37], and were probably previously exposed to environmental antigens. In healthy adults, CD8 + T cells are higher among those living in urban areas of Quebec, Canada [38]. We did not analyze the phenotype of these cells, but in animals, CD8 + memory T cells inhibit allergic inflammation after exposure to environmental antigens or infections [39]. Conversely, in adult asthma, CD8 + T-cell density has been predictive of lung function decline [40].

In ASM, the mast cell density was the highest, as expected in asthma [41]. Tryptase and chymase + mast cells are the most common mast cell types, and differences in their airway wall microlocation and functional properties may affect asthma pathogenesis, although few studies have analyzed both of these mast cell phenotypes in pediatric asthma. Here, we report that while tryptase + mast cell density correlated positively with BM thickness, chymase + count was positively correlated with ACT and quality-of-life score. Other studies have reported that chymase + mast cells have a protective role in severely asthmatic adults [42], and we show here for the first time a similar positive association in children with STRA. It is possible that chymase-induced IL33 modulation accounts for the beneficial effects in severe asthma [43].

In our study, BM thickness was similar to that described in a study comparing airway remodeling in children with STRA with that in control subjects [7]. Moreover, BM thickness was positively correlated with blood eosinophil density and sputum IL-4 level, which are biomarkers of eosinophilic allergic asthma and the T2 endotype, respectively. Remodeling, which includes thickening of the BM as well as other airway wall compartments, seems to occur early in infancy [44]. However, as in a study by Bossley et al. [7], we did not find it to be correlated with lung function, suggesting that although it is a very characteristic pathological feature of asthma, micrometric RBM thickening does not significantly impact airway mechanics.

Contrary to this study, in adults with STRA asthma of São Paulo, another study found no correlation between inflammatory cells and asthma control and obstruction [29]. Possibly due to the remodeling present in the adult cases, it found correlations with ASM thickness only. These data indicate that in children, there is a longer window of time for therapeutic interventions that may modify the disease course.

This study has several limitations. For ethical reasons, we did not have a control group of healthy individuals or those with mild/moderate asthma, which limited the data interpretation. Moreover, this was a single-center study, so the number of patients with STRA was limited. Due to difficulties in obtaining adequate samples from all patients, the sample number was not the same for all analyses, which limited our correlation analyses. Biopsies were often small, so not all airway wall layers, such as ASM, were fully represented in terms of different markers and structures. Nevertheless, such a broad representation of airway compartments has been performed in very few studies. This is also the first South American study characterizing STRA in school children and adolescents, and the genetic, environmental, and socioeconomic background of participants were likely to be significantly different from those in northern countries.

In conclusion, we characterized the inflammatory profile of a Brazilian cohort of urban schoolchildren and adolescents with STRA by invasive and noninvasive methods. We showed that besides eosinophils, other cell types at different airway (micro)locations were correlated with asthma control and lung function. Namely, submucosal and BAL neutrophil densities correlated with worsened lung function, but intraepithelial neutrophils were positively correlated with sGaw. Bronchial CD8 + T cells, abundant in bronchial biopsies, showed protective associations with asthma control and sputum cytokine levels. This profile is probably the result of genetic and environmental conditions and might reflect a transition to adult severe asthma. It is not known whether the persistence of these cells in the airways throughout adulthood will affect lung health, but they might be associated with the development of fixed obstruction and accelerated lung function decline [40].

## Supplementary Information

**Additional file 1: Figure S1.** Flow diagram of study design and procedures. **Table S1.** Antibodies used in the study. **Table S2: **Relationship between number of cells per compartment in bronchial biopsies of patients with STRA (Intraepithelial – IE, Submucosal – SM and Airway smooth muscle – ASM) with bronchoalveolar lavage (BALF) (n=11). **Table S3.** Significative correlation between bronchoalveolar lavage (BALF) cytology and cytokines and lung function. **Table S4.** Results of bronchoalveolar lavage (BAL) cultures, virus and cellularity. **Table S5. **Relationship between number of cells per compartment (Intraepithelial – IE, Submucosal – SM and Airway smooth muscle – ASM) with pulmonary function test in bronchial biopsies of patients with STRA (n=11). **Table S6**. Relationship between number of cells per compartment (Intraepithelial – IE, Submucosal – SM and Airway smooth muscle – ASM) with follow-up data and assessment of inflammation in bronchial biopsies of patients with STRA (n=11). **Figure S2. **Correlation between CD8 T cells in bronchial biopsies and sputum interleukins in the patients with STRA (n=11). **Figure S3**. Heatmap of correlation among cytokine levels in the sputum, bronchoalveolar lavage (BAL), and plasma. The correlation coefficients are color coded from deep red (−1) to deep blue (1).

## Abbreviations

ACT: Asthma control test
ASM: Airway smooth muscle
ATS: American Thoracic Society
BAL: Bronchoalveolar lavage
BD: Bronchodilator
BM: Basement membrane
BMI: Body mass index
ERS: European Respiratory Society
FEF 25–75%: Forced expiratory flow at 25–75% of FVC
FENO: Fractional exhaled nitric oxide
FEV1: Forced expiratory volume in the first second
FVC: Forced vital capacity
GINA: Global Initiative for Asthma
IE: Intraepithelial
IgE: Immunoglobulin E
IL: Interleukins
LABA: Long-acting β2 agonist
LTRA: Leukotriene receptor antagonist
pAQLQ: Pediatric asthma quality-of-life questionnaire
RV: Residual volume
sGAW: Airway conductance
SM: Submucosal
STRA: Severe treatment-resistant asthma
TLC: Total lung capacity
