# Supplementary material for: Bronchial eosinophils, neutrophils, and CD8 + T cells influence asthma control and lung function in schoolchildren and adolescents with severe treatment-resistant asthma
Source: Respir Res. 2022 Dec 9;23:335. doi: 10.1186/s12931-022-02259-4 (PMC9733356; doi:10.1186/s12931-022-02259-4)
Supplement: Supplementary file 1 — Additional file 1: Figure S1. Flow diagram of study design and procedures. Table S1. Antibodies used in the study. Table S2: Relationship between number of cells per compartment in bronchial biopsies of patients with STRA (Intraepithelial – IE, Submucosal – SM and Airway smooth muscle – ASM) with bronchoalveolar lavage (BALF) (n=11). Table S3. Significative correlation between bronchoalveolar lavage (BALF) cytology and cytokines and lung function. Table S4. Results of bronchoalveolar lavage (BAL) cultures, virus and cellularity. Table S5. Relationship between number of cells per compartment (Intraepithelial – IE, Submucosal – SM and Airway smooth muscle – ASM) with pulmonary function test in bronchial biopsies of patients with STRA (n=11). Table S6. Relationship between number of cells per compartment (Intraepithelial – IE, Submucosal – SM and Airway smooth muscle – ASM) with follow-up data and assessment of inflammation in bronchial biopsies of patients with STRA (n=11). Figure S2. Correlation between CD8 T cells in bronchial biopsies and sputum interleukins in the patients with STRA (n=11). Figure S3. Heatmap of correlation among cytokine levels in the sputum, bronchoalveolar lavage (BAL), and plasma. The correlation coefficients are color coded from deep red (−1) to deep blue (1). [file 12931_2022_2259_MOESM1_ESM.docx]

**Additional file**

**Bronchial eosinophils, neutrophils, and CD8+ T cells influence asthma control and lung function in schoolchildren and adolescents with severe treatment-resistant asthma**

Authors: Miriam Cardoso Neves Eller^1^ MD, MSc; Karina Pierantozzi Vergani^1^ MD, MSc; Beatriz Mangueira Saraiva-Romanholo^2^ PhD; Natália de Souza Xavier Costa^3^ PhD; Jôse Mára de Brito^3,6^ PhD; Leila Antonangelo^4,5^ MD, PhD; Caroline Silvério Faria^5^ MSc; Joaquim Carlos Rodrigues^1^ MD, PhD; Thais Mauad^3^ MD, PhD

^1^ Unidade de Pneumologia Pediátrica, Instituto da Criança, Hospital das Clínicas HCFMUSP, Faculdade de Medicina, Universidade de São Paulo, São Paulo,SP, BR

^2^ Universidade da Cidade de São Paulo (UNICID), São Paulo, SP, BR

^3^ Departamento de Patologia, Faculdade de Medicina, Universidade de São Paulo, SP, BR

^4^ Divisao de Patologia Clinica- Departamento de Patologia, Hospital das Clinicas HCFMUSP, Faculdade de Medicina, Universidade de São Paulo, São Paulo,SP, BR

^5^ Laboratorio de Investigacao Clinica (LIM03), Faculdade de Medicina, Universidade de São Paulo, SP, BR

^6^ Departamento de Patologia e Parasitologia – Universidade Federal de Alfenas, Alfenas, MG, BR

Corresponding author: Miriam Cardoso Neves Eller, *Instituto da Criança, HCFMUSP*. Mailing address Rua Muniz de Sousa, 1020, apt 132, CEP 01534-000, São Paulo, SP, Brazil.

Email: [miriamneves@gmail.com](mailto:miriamneves@gmail.com)

**METHODS**


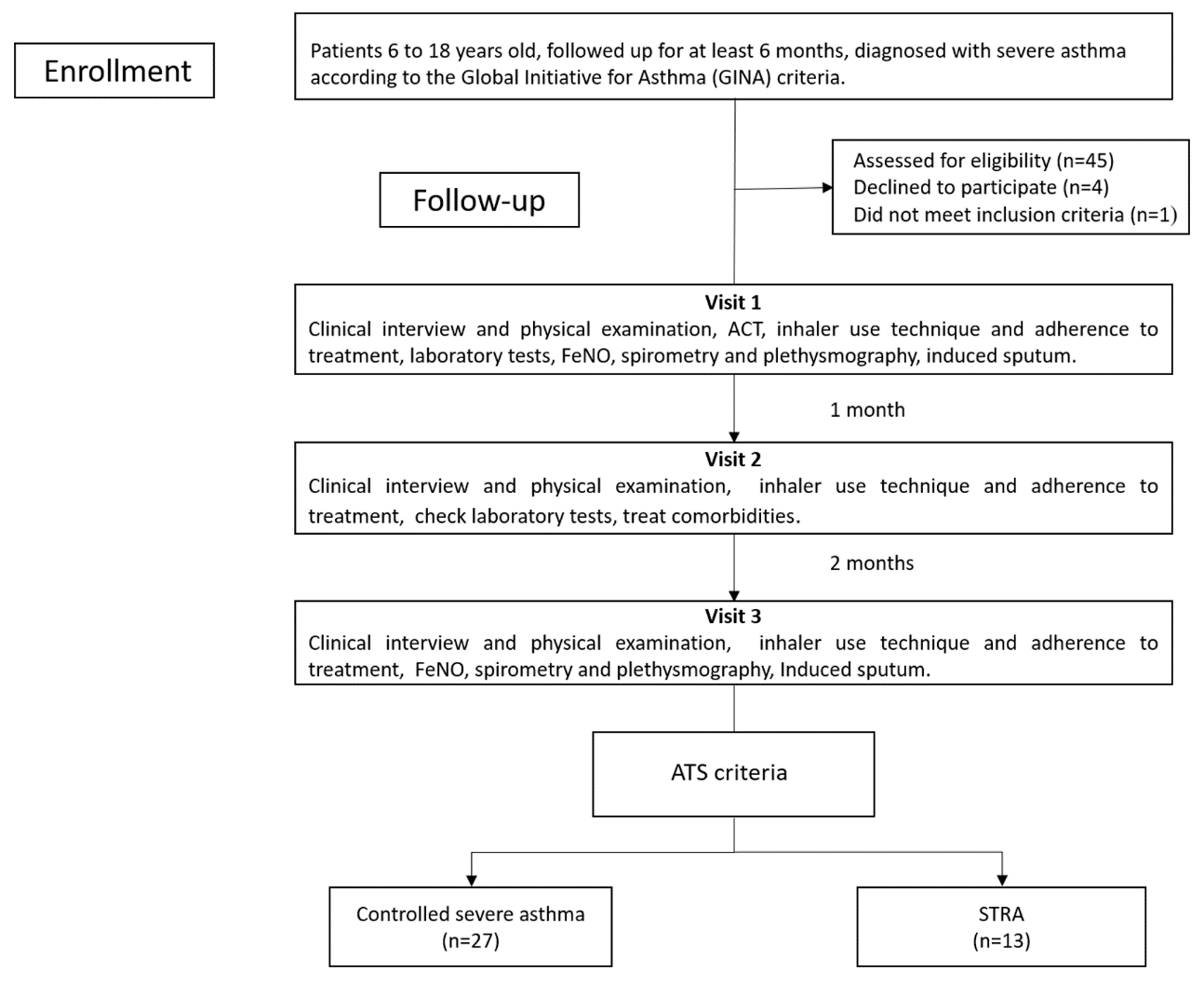


**Figure S1.** Flow diagram of study design and procedures

**Bronchial tissue processing and immunohistochemistry.**

**Table S I.** Antibodies used in the study

| **Antibody** | **Dilution** | **Antigen retrieval** | **Cod.** | **Manufacturer** |
| --- | --- | --- | --- | --- |
| **CD4** | 1:75 | High-temperature: pH6 citrate buffer | NCL-L-368 | Leica Biosystems Newcastle Ltd, UK |
| **CD8** | 1:400 | High-temperature: pH6 citrate buffer | M7103 | Dako, Glostrup, Denmark |
| **Tryptase** | 1:8000 | High-temperature: pH6 citrate buffer | M705 | Dako, Glostrup, Denmark |
| **Chymase** | 1:1000 | High-temperature: pH6 citrate buffer | NCL-MCC | Dako, Glostrup, Denmark |
| **Elastase** | 1:100 | Without antigen retrieval | M0752 | Dako, Glostrup, Denmark |

**RESULTS**

| **BALF** | | **Thickness MB (µm)** | | **IE (cell/mm BM)** | | | | | | **SM (cell/mm BM)** | | | | **ASM (cell/mm2 area)** | | | | | | | | |
| --- | --- | --- | --- | --- | --- | --- | --- | --- | --- | --- | --- | --- | --- | --- | --- | --- | --- | --- | --- | --- | --- | --- |
|  |  |  |  | **Tryptase** | | **Chymase** | | **CD4** | | **Neutrophils** | | **CD8** | | **Eosinophils** | | **Neutrophils** | | **CD4** | | **CD8** | |  |
|  |  | **r** | **p** | **r** | **p** | **R** | **p** | **r** | **p** | **r** | **p** | **r** | **P** | **r** | **p** | **r** | **p** | **r** | **p** | **r** | **p** |  |
| **Inflammatory cells** | |  |  |  |  |  |  |  |  |  |  |  |  |  |  |  |  |  |  |  |  |  |
|  | Neutrophils (%) | -0.600 | 0.067 | -0.450 | 0.224 | **-0.883** | **0.002** | 0.433 | 0.244 | 0.582 | 0.06 | -0.333 | 0.347 | 0.086 | 0.872 | -0.143 | 0.76 | 0.200 | 0.704 | -0.464 | 0.294 |  |
|  | Eosinophils (%) | 0.603 | 0.065 | 0.303 | 0.429 | 0.094 | 0.811 | -0.255 | 0.507 | 0.110 | 0.747 | **0.671** | **0.034** | **0.883** | **0.020** | 0.473 | 0.284 | 0.088 | 0.868 | **0.775** | **0.041** |  |
|  | Basophils (%) | **0.659** | **0.038** | 0.298 | 0.436 | 0.274 | 0.476 | -0.365 | 0.334 | 0.256 | 0.448 | 0.541 | 0.106 | 0.655 | 0.158 | **0.802** | **0.030** | 0.131 | 0.805 | 0.535 | 0.216 |  |
|  | Lymphocytes (%) | 0.219 | 0.544 | 0.326 | 0.391 | **0.828** | **0.006** | -0.059 | 0.881 | **-0.679** | **0.022** | 0.097 | 0.789 | -0.143 | 0.787 | 0.000 | 1.000 | 0.145 | 0.784 | 0.072 | 0.878 |  |
| **Cytokines** | |  |  |  |  |  |  |  |  |  |  |  |  |  |  |  |  |  |  |  |  |  |
|  | IL-6 | -0.591 | 0.072 | -0.077 | 0.845 | -0.227 | 0.557 | **0.706** | **0.034** | -0.009 | 0.978 | -0.257 | 0.473 | 0.143 | 0.787 | -0.036 | 0.939 | 0.493 | 0.321 | -0.291 | 0.527 |  |
|  | IL-7 | -0.067 | 0.855 | -0.467 | 0.205 | 0.150 | 0.700 | -0.067 | 0.865 | -0.045 | 0.894 | -0.079 | 0.829 | 0.543 | 0.266 | 0.107 | 0.819 | **0.886** | **0.019** | -0.286 | 0.535 |  |
|  | IL-12 | -0.338 | 0.340 | **-0.766** | **0.016** | -0.136 | 0.728 | -0.170 | 0.663 | 0.095 | 0.780 | 0.058 | 0.873 | 0.551 | 0.257 | 0.185 | 0.691 | 0.543 | 0.266 | -0.037 | 0.937 |  |
|  | IL-13 | -0.129 | 0.722 | **-0.780** | **0.013** | 0.042 | 0.915 | -0.269 | 0.484 | 0.023 | 0.946 | -0.069 | 0.850 | 0.441 | 0.381 | 0.356 | 0.434 | 0.377 | 0.461 | 0.074 | 0.875 |  |
|  | IL-17 | -0.406 | 0.244 | **-0.767** | **0.016** | -0.050 | 0.898 | -0.183 | 0.637 | 0.123 | 0.719 | 0.012 | 0.973 | 0.486 | 0.329 | 0.144 | 0.758 | 0.600 | 0.208 | -0.180 | 0.699 |  |
|  | GMCSF | -0.176 | 0.627 | **-0.833** | **0.005** | 0.083 | 0.831 | **-0.700** | **0.036** | 0.282 | 0.401 | 0.370 | 0.293 | 0.371 | 0.468 | 0.286 | 0.535 | 0.086 | 0.872 | 0.214 | 0.645 |  |
|  | IFN-γ | 0.006 | 0.987 | **-0.700** | **0.036** | 0.333 | 0.381 | -0.650 | 0.058 | 0.164 | 0.631 | 0.321 | 0.365 | 0.257 | 0.623 | 0.357 | 0.432 | 0.200 | 0.704 | 0.179 | 0.702 |  |

**Table S II:** Relationship between number of cells per compartment in bronchial biopsies of patients with STRA (Intraepithelial – IE, Submucosal – SM and Airway smooth muscle – ASM) with bronchoalveolar lavage (BALF) (n=11).

IL: Interleukin; GMCSF: Granulocyte-macrophage colony-stimulating factor; IFN-γ: Interferon gama; MCP1: Monocyte Chemoattractant Protein-1.

**Table S III:** Significative correlation between bronchoalveolar lavage (BALF) cytology and cytokines and lung function.

| **BALF** | **Pre-BD FEF25-75 (Pred %)** | | **Pre-BD RV/TLC (%)** | | **Pre-BD FEV1 (Pred %)** | | **Pre-BD FEV1/SVC** | | **Pre-BD TLC (%)** | | **Post-BD RV/TCL (%)** | | **Post-BD FEV1/FCV** | | **Post-BD FEF25-75 (Pred %)** | | **Post-BD FEV1**  **(Pred %)** | | **Post-BD TLC (%)** | | **Post-BD RV (%)** | |
| --- | --- | --- | --- | --- | --- | --- | --- | --- | --- | --- | --- | --- | --- | --- | --- | --- | --- | --- | --- | --- | --- | --- |
|  | **r** | **p** | **r** | **p** | **r** | **p** | **r** | **p** | **r** | **p** | **r** | **p** | **r** | **p** | **r** | **p** | **r** | **p** | **r** | **p** | **r** | **p** |
| Total cells (mm3) | 0.333 | 0.347 | -0.345 | 0.328 | 0.067 | 0.855 | 0.030 | 0.934 | **-0.683^*^** | **0.042** | -0.400 | 0.286 | -0.176 | 0.627 | 0.164 | 0.651 | 0.091 | 0.803 | -0.238 | 0.570 | -0.310 | 0.456 |
| Neutrophils (%) | -0.335 | 0.263 | 0.165 | 0.590 | -0.363 | 0.223 | -0.412 | 0.162 | -0.109 | 0.737 | 0.238 | 0.457 | -0.462 | 0.112 | **-0.582^*^** | **0.037** | **-0.566^*^** | **0.044** | -0.374 | 0.258 | -0.009 | 0.979 |
| Basophils (%) | **-0.636^*^** | **0.019** | 0.487 | 0.091 | **-0.663^*^** | **0.013** | **-0.568^*^** | **0.043** | 0.097 | 0.765 | 0.362 | 0.247 | -0.392 | 0.185 | -0.487 | 0.091 | **-0.582^*^** | **0.037** | 0.453 | 0.162 | 0.452 | 0.163 |
| Lymphocytes (%) | 0.509 | 0.076 | -0.358 | 0.230 | **0.553^*^** | **0.050** | 0.547 | 0.053 | -0.111 | 0.732 | -0.301 | 0.341 | **0.696^**^** | **0.008** | **0.746^**^** | **0.003** | **0.740^**^** | **0.004** | 0.055 | 0.873 | -0.132 | 0.699 |
| IL6 | 0.218 | 0.475 | -0.307 | 0.308 | 0.215 | 0.481 | 0.128 | 0.676 | -0.302 | 0.340 | -0.035 | 0.913 | 0.226 | 0.458 | 0.086 | 0.779 | 0.100 | 0.744 | **-0.659^*^** | **0.027** | -0.189 | 0.579 |
| IL7 | -0.214 | 0.482 | 0.187 | 0.541 | -0.071 | 0.817 | -0.038 | 0.901 | 0.130 | 0.688 | **0.678^*^** | **0.015** | 0.060 | 0.845 | -0.258 | 0.394 | -0.253 | 0.405 | -0.219 | 0.518 | 0.582 | 0.060 |
| IL10 | -0.387 | 0.191 | 0.376 | 0.206 | -0.269 | 0.375 | -0.040 | 0.896 | 0.287 | 0.366 | **0.595^*^** | **0.041** | -0.078 | 0.800 | -0.344 | 0.250 | -0.298 | 0.323 | -0.040 | 0.908 | 0.591 | 0.056 |
| IL13 | -0.218 | 0.474 | 0.405 | 0.170 | -0.076 | 0.804 | 0.071 | 0.818 | 0.189 | 0.556 | **0.663^*^** | **0.019** | -0.037 | 0.905 | -0.286 | 0.344 | -0.258 | 0.396 | -0.152 | 0.655 | 0.556 | 0.075 |
| IL17 | -0.331 | 0.269 | 0.293 | 0.332 | -0.221 | 0.468 | -0.110 | 0.719 | 0.070 | 0.828 | **0.627^*^** | **0.029** | -0.149 | 0.627 | -0.420 | 0.153 | -0.392 | 0.185 | -0.314 | 0.346 | 0.473 | 0.142 |
| GCSF | 0.177 | 0.563 | -0.149 | 0.627 | 0.210 | 0.491 | 0.177 | 0.563 | -0.265 | 0.406 | 0.186 | 0.564 | 0.254 | 0.402 | 0.033 | 0.914 | 0.039 | 0.900 | **-0.651^*^** | **0.030** | -0.059 | 0.863 |
| GMCSF | **-0.582^*^** | **0.037** | **0.566^*^** | **0.044** | -0.456 | 0.117 | -0.198 | 0.517 | 0.490 | 0.106 | **0.664^*^** | **0.018** | -0.385 | 0.194 | **-0.604^*^** | **0.029** | -0.538 | 0.058 | 0.114 | 0.739 | **0.618^*^** | **0.043** |
| IFN-γ | **-0.560^*^** | **0.046** | **0.566^*^** | **0.044** | -0.423 | 0.150 | -0.176 | 0.566 | 0.504 | 0.094 | **0.720^**^** | **0.008** | -0.231 | 0.448 | -0.527 | 0.064 | -0.473 | 0.103 | 0.114 | 0.739 | **0.709^*^** | **0.015** |

IL: Interleukin; GCSF: Granulocyte colony stimulating factor; GMCSF: Granulocyte-macrophage colony-stimulating factor; IFN-γ: Interferon gama, BD: bronchodilator; FEV1: forced expiratory volume in the first second; FEF 25-75%: forced expiratory flow at 25-75% of FVC; RV/TLC: ratio of residual volume to total lung capacity; FEV1/SVC: ratio of forced expiratory volume in the first second to slow vital capacity; TLC: total lung capacity; ; FEV1/SVC: ratio of forced expiratory volume in the first second to slow vital capacity; RV/TLC: ratio of residual volume to total lung capacity; RV: residual volume.

**Table S IV:** Results of bronchoalveolar lavage (BAL) cultures, virus and cellularity.

| **Patient** | **Culture** | **BAAR** | **Fungus** | **Virus** | **Neutrophils (%)** | **Eosinophils (%)** | **Basophils (%)** | **Lymphocytes (%)** | **Monocytes (%)** | **Macrophages (%)** | **Epithelial cells (%)** |
| --- | --- | --- | --- | --- | --- | --- | --- | --- | --- | --- | --- |
| 1 | Negative | Negative | Negative | Negative | 3 | 7 | 14 | 4 | 0 | 60 | 25 |
| 2 | Negative | Negative | Negative | Coronavirus NL65 | 0 | 0 | 0 | 5 | 0 | 4 | 90 |
| 3 | S. pneumonie | Negative | Negative | Negative | 22 | 2 | 0 | 0 | 0 | 60 | 16 |
| 4 | Negative | Negative | Negative | Negative | 3 | 3 | 0 | 8 | 0 | 10 | 75 |
| 5 | Polimicrobial | Negative | Negative | Negative | 0 | 0 | 0 | 3 | 0 | 1 | 96 |
| 6 | Polimicrobial | Negative | Negative | Negative | 13 | 66 | 0 | 4 | 1 | 6 | 10 |
| 7 | Polimicrobial | Negative | Aspergillus | Negative | 82 | 0 | 0 | 2 | 1 | 10 | 5 |
| 8 | Polimicrobial | Negative | Negative | Negative | 1 | 1 | 0 | 23 | 1 | 24 | 51 |
| 9 | Polimicrobial | Negative | Negative | Negative | 48 | 0 | 0 | 2 | 1 | 45 | 5 |
| 10 | Polimicrobial | Negative | Negative | Negative | 96 | 1 | 0 | 1 | 0 | 2 | 0 |
| 11 | Negative | Negative | Negative | Negative | 12 | 0 | 0 | 18 | 0 | 5 | 65 |
| 12 | Polimicrobial | Negative | Negative | Negative | 45 | 18 | 1 | 0 | 0 | 28 | 6 |
| 13 | Polimicrobial | Negative | Negative | Negative | 13 | 3 | 0 | 18 | 0 | 40 | 23 |

Search for 12 respiratory viruses, Mycoplasma pneumoniae, Chlamydophila pneumoniae, and Bordetella pertussis, was performed by multiplex real-time PCR assay FilmArray® Respiratory Panel (BioFire, Utah, US).

BAAR: Alcohol-acid resistant bacillus

**Table S V.** Relationship between number of cells per compartment (Intraepithelial – IE, Submucosal – SM and Airway smooth muscle – ASM) with pulmonary function test in bronchial biopsies of patients with STRA (n=11).

|  | **Thickness MB** | | **IE (cell/mm BM)** | | | | | | | | **SM (cell/mm BM)** | | | | | | | | **ASM (cell/mm2 area)** | |
| --- | --- | --- | --- | --- | --- | --- | --- | --- | --- | --- | --- | --- | --- | --- | --- | --- | --- | --- | --- | --- |
| **Pulmonary Function Test** | **(µm)** | | **Eosinophils** | | **Neutrophils** | | **CD4** | | **CD4/CD8** | | **Eosinophils** | | **Neutrophils** | | **Tryptase** | | **CD4/CD8** | | **CD4** | |
|  | **r** | **P** | **r** | **p** | **r** | **p** | **r** | **p** | **r** | **p** | **r** | **p** | **r** | **p** | **r** | **p** | **r** | **p** | **r** | **P** |
| FEV1 variation pós-BD(%) | 0.261 | 0.467 | **0.720** | **0.013** | 0.517 | 0.154 | 0.333 | 0.381 | 0.357 | 0.432 | **0.645** | **0.032** | 0.282 | 0.401 | **0.806** | **0.005** | -0.583 | 0.099 | 0.600 | 0.208 |
| Pre-BD FEF25-75 | -0.455 | 0.187 | -0.542 | 0.085 | -0.217 | 0.576 | 0.217 | 0.576 | 0.429 | 0.337 | -0.582 | 0.060 | **-0.645** | **0.032** | -0.333 | 0.347 | -0.083 | 0.831 | -0.771 | 0.072 |
| Pre-BD RV/TLC (%) | 0.442 | 0.200 | 0.305 | 0.361 | 0.467 | 0.205 | **-0.700** | **0.036** | -0.571 | 0.180 | 0.145 | 0.670 | 0.491 | 0.125 | 0.115 | 0.751 | -0.283 | 0.460 | 0.143 | 0.787 |
| Pre-BD FEV1 | -0.345 | 0.328 | -0.560 | 0.073 | -0.133 | 0.732 | 0.067 | 0.865 | 0.286 | 0.535 | **-0.709** | **0.015** | **-0.755** | **0.007** | -0.382 | 0.276 | 0.067 | 0.865 | -0.657 | 0.156 |
| Pre-BD FEV1/SVC | -0.467 | 0.174 | -0.487 | 0.128 | -0.330 | 0.932 | -0.183 | 0.637 | 0.393 | 0.383 | **-0.664** | **0.026** | -0.636 | **0.035** | -0.455 | 0.187 | 0.133 | 0.732 | **-0.886** | **0.019** |
| Pre-BD RV | 0.515 | 0.128 | 0.314 | 0.346 | 0.417 | 0.265 | **-0.683** | **0.042** | -0.643 | 0.119 | 0.182 | 0.593 | 0.518 | 0.102 | 0.152 | 0.676 | -0.167 | 0.668 | 0.143 | 0.787 |
| Pre-BD sGawPré | -0.333 | 0.347 | -0.547 | 0.082 | **0.667^*^** | **0.005** | -0.017 | 0.966 | 0.179 | 0.702 | -0.473 | 0.142 | -0.573 | 0.066 | **-0.661** | **0.038** | 0.600 | 0.088 | -0.486 | 0.329 |
| Pre-BD Raw | 0.267 | 0.455 | 0.192 | 0.572 | 0.243 | 0.529 | 0.100 | 0.797 | -0.414 | 0.355 | 0.305 | 0.361 | **0.702** | **0.016** | 0.359 | 0.309 | -0.494 | 0.177 | 0.725 | 0.103 |
| Pos-BD VEF1 | -0.176 | 0.627 | -0.146 | 0.669 | 0.200 | 0.606 | 0.217 | 0.576 | 0.464 | 0.294 | -0.327 | 0.326 | **-0.700** | **0.016** | 0.164 | 0.651 | -0.300 | 0.433 | -0.314 | 0.544 |
| Pos-BD RV/TCL | 0.367 | 0.332 | 0.182 | 0.614 | 0.357 | 0.385 | **-0.683** | **0.042** | -0.679 | 0.094 | 0.006 | 0.987 | 0.200 | 0.58 | -0.150 | 0.700 | 0.405 | 0.320 | 0.314 | 0.544 |
| Pos-BD FEF25-75 | -0.127 | 0.726 | -0.191 | 0.573 | 0.133 | 0.732 | 0.200 | 0.606 | 0.286 | 0.535 | -0.373 | 0.259 | -0.764 | **0.006** | 0.115 | 0.751 | -0.117 | 0.765 | -0.200 | 0.704 |
| Pos-BD FEV1 | -0.176 | 0.627 | -0.146 | 0.669 | 0.200 | 0.606 | 0.217 | 0.576 | 0.464 | 0.294 | -0.327 | 0.326 | **-0.700** | **0.016** | 0.164 | 0.651 | -0.300 | 0.433 | -0.314 | 0.544 |
| Pos-BD FEV1/SVC | 0.006 | 0.987 | 0.114 | 0.739 | 0.217 | 0.576 | 0.067 | 0.865 | 0.179 | 0.702 | -0.127 | 0.709 | **-0.682** | **0.021** | 0.127 | 0.726 | 0 | 1 | -0.200 | 0.704 |
| Pos-BD RV | 0.550 | 0.125 | 0.340 | 0.336 | 0.476 | 0.233 | **-0.717** | **0.003** | **-0.857** | **0.014** | 0.115 | 0.751 | 0.261 | 0.467 | 0.083 | 0.831 | 0.310 | 0.456 | 0.314 | 0.544 |

Functional pulmonary data are expressed as the mean percentages of predictions and standard deviations according to the Global Lung Function Initiative reference values for spirometry and Polgar and Weng’s reference values for plethysmography. Abbreviations: BD: bronchodilator; FEV1: forced expiratory volume in the first second; FVC: forced vital capacity; FEV1/SVC: ratio of forced expiratory volume in the first second to slow vital capacity; FEF 25-75%: forced expiratory flow at 25-75% of FVC; TLC: total lung capacity; RV: residual volume; RV/TLC: ratio of residual volume to total lung capacity

**Table S VI.** Relationship between number of cells per compartment (Intraepithelial – IE, Submucosal – SM and Airway smooth muscle – ASM) with sputum in bronchial biopsies of patients with STRA (n=11).

| **Sputum** | **Thickness MB (µm)** | | **IE (cell/mm BM)** | | | | | | **SM (cell/mm BM)** | | | | | | **ASM (cell/mm2 area)** | | | |
| --- | --- | --- | --- | --- | --- | --- | --- | --- | --- | --- | --- | --- | --- | --- | --- | --- | --- | --- |
|  |  |  | **Eosinophils** | | **Chymase** | | **CD4** | | **Eosinophils** | | **Neutrophils** | | **CD8** | | **Neutrophils** | | **Tryptase** | |
|  | **r** | **p** | **r** | **p** | **r** | **p** | **R** | **p** | **r** | **p** | **r** | **p** | **r** | **p** | **r** | **p** | **r** | **p** |
| IL-1β | 0.500 | 0.391 | **-0.810** | **0.015** | **-0.821** | **0.023** | 0.464 | 0.294 | -0.500 | 0.207 | 0.381 | 0.352 | **-0.786** | **0.036** | 1.000 | 0.000 | -0.400 | 0.505 |
| IL-2 | 0.359 | 0.553 | -0.634 | 0.091 | -0.185 | 0.691 | 0.185 | 0.691 | -0.647 | 0.083 | -0.076 | 0.858 | **-0.906** | **0.005** | -0.224 | 0.718 | -0.783 | 0.118 |
| IL-4 | **0.894** | **0.041** | -0.464 | 0.247 | -0.535 | 0.216 | **0.757** | **0.049** | -0.218 | 0.604 | 0.355 | 0.389 | -0.709 | 0.074 | 0.112 | 0.858 | -0.224 | 0.718 |
| IL-5 | 0.700 | 0.188 | -0.699 | 0.054 | -0.198 | 0.670 | 0.306 | 0.504 | -0.687 | 0.060 | -0.169 | 0.690 | **-0.837** | **0.019** | 0.051 | 0.935 | -0.821 | 0.089 |
| IL-7 | 0.500 | 0.391 | -0.443 | 0.272 | -0.429 | 0.337 | 0.464 | 0.294 | -0.192 | 0.649 | 0.036 | 0.933 | **-0.793** | **0.033** | 0.000 | 1.000 | -0.300 | 0.624 |
| IL-10 | 0.600 | 0.285 | -0.671 | 0.069 | -0.321 | 0.482 | 0.500 | 0.253 | -0.419 | 0.301 | 0.108 | 0.799 | **-0.775** | **0.041** | -0.667 | 0.219 | -0.821 | 0.089 |
| IL-12 | 0.200 | 0.747 | -0.395 | 0.333 | 0.090 | 0.848 | -0.054 | 0.908 | -0.335 | 0.417 | -0.335 | 0.417 | **-0.883** | **0.008** | -0.500 | 0.391 | -0.600 | 0.285 |
| IL-13 | 0.000 | 1.000 | -0.479 | 0.230 | 0.378 | 0.403 | -0.090 | 0.848 | -0.503 | 0.204 | **-0.731** | **0.040** | -0.523 | 0.229 | 0.000 | 1.000 | **-0.900** | **0.037** |
| IL-17 | 0.600 | 0.285 | **-0.881** | **0.004** | -0.607 | 0.148 | 0.571 | 0.180 | -0.500 | 0.207 | 0.071 | 0.867 | **-0.929** | **0.003** | **-0.900** | **0.037** | -0.700 | 0.188 |
| GCSF | 0.600 | 0.285 | **-0.762** | **0.028** | -0.571 | 0.180 | 0.286 | 0.535 | -0.571 | 0.139 | 0.143 | 0.736 | **-0.964** | **0.000** | -0.600 | 0.285 | -0.700 | 0.188 |
| GMCSF | 0.500 | 0.391 | -0.429 | 0.289 | -0.250 | 0.589 | **0.786** | **0.036** | 0.000 | 1.000 | 0.262 | 0.531 | -0.571 | 0.180 | -0.600 | 0.285 | -0.700 | 0.188 |
| IFN-γ | 0.700 | 0.188 | **-0.905** | **0.002** | -0.714 | 0.071 | 0.536 | 0.215 | -0.595 | 0.120 | 0.190 | 0.651 | **-0.964** | **0.000** | **-0.900** | **0.037** | -0.700 | 0.188 |
| MCP1 | 0.300 | 0.624 | -0.690 | 0.058 | -0.321 | 0.482 | 0,000 | 1.000 | **-0.762** | **0.028** | -0.048 | 0.911 | **-0.929** | **0.003** | -0.600 | 0.285 | -0.800 | 0.104 |
| MIP-1b | **0.900** | **0.037** | **-0.929** | **0.001** | -0.429 | 0.337 | 0.429 | 0.337 | -0.524 | 0.183 | -0.095 | 0.823 | -0.714 | 0.071 | -0.500 | 0.391 | -0.700 | 0.188 |
| TNF-α | 0.500 | 0.391 | -0.643 | 0.086 | **-0.821** | **0.023** | 0.464 | 0.294 | -0.310 | 0.456 | 0.524 | 0.183 | **-0.893** | **0.007** | -0.700 | 0.188 | 0.100 | 0.873 |

IL-1β: Interleukin-1 beta; IL: Interleukin; GCSF: Granulocyte colony stimulating factor; GMCSF: Granulocyte-macrophage colony-stimulating factor; IFN-γ: Interferon gama; MCP1: Monocyte Chemoattractant Protein-1; MIP-1b: macrophage inflammatory protein 1 beta; TNF-α: Tumor necrosis factor alpha.

**Table S VII**. Relationship between number of cells per compartment (Intraepithelial – IE, Submucosal – SM and Airway smooth muscle – ASM) with follow-up data and assessment of inflammation in bronchial biopsies of patients with STRA (n=11)

| **Variables** | | **Thickness MB (µm)** | | **IE (cell/mm BM)** | | | | | | **SM (cell/mm BM)** | | | | **ASM (cell/mm2 area)** | | | | | |
| --- | --- | --- | --- | --- | --- | --- | --- | --- | --- | --- | --- | --- | --- | --- | --- | --- | --- | --- | --- |
|  |  |  |  | **Quimase** | | **CD8** | | **CD4/CD8 ratio** | | **CD8 SM** | | **CD4/CD8 ratio** | | **Neutrophils** | | **Chymase** | | **CD8** | |
|  |  | **R** | **p** | **R** | **p** | **r** | **P** | **r** | **p** | **r** | **p** | **r** | **p** | **r** | **p** | **r** | **p** | **r** | **p** |
| **Follow-up data** | |  |  |  |  |  |  |  |  |  |  |  |  |  |  |  |  |  |  |
|  | Age | 0.442 | 0.200 | 0.167 | 0.668 | 0.405 | 0.320 | **-0.786** | **0.036** | -0.018 | 0.96 | 0.233 | 0.546 | 0 | 1 | 0.071 | 0.879 | -0.500 | 0.253 |
|  | pAQLQ | 0.152 | 0.676 | -0.200 | 0.606 | 0.476 | 0.233 | -0.714 | 0.071 | -0.576 | 0.082 | 0.467 | 0.205 | -0.429 | 0.337 | **0.857** | **0.014** | -0.429 | 0.337 |
|  | ACT | 0.618 | 0.057 | 0.269 | 0.484 | **0.826** | **0.011** | **-0.821** | **0.023** | -0.287 | 0.421 | 0.168 | 0.666 | 0 | 1 | **0.919** | **0.003** | 0.073 | 0.877 |
| **Assessment of inflammation** | |  |  |  |  |  |  |  |  |  |  |  |  |  |  |  |  |  |  |
|  | FeNo (ppb) | 0.535 | 0.111 | **0.828** | **0.006** | 0.587 | 0.126 | -0.631 | 0.129 | 0.498 | 0.143 | 0.059 | 0.881 | 0.643 | 0.119 | 0.595 | 0.159 | 0.288 | 0.531 |
|  | Serum leucocytes/mm^3^ | 0.248 | 0.489 | 0.033 | 0.932 | 0.571 | 0.139 | -0.536 | 0.215 | -0.212 | 0.556 | **0.667** | **0.050** | 0.214 | 0.645 | 0.679 | 0.094 | -0.357 | 0.432 |
|  | Serum eosinophils (%) | **0.772** | **0.009** | 0.435 | 0.242 | 0.595 | 0.120 | **-0.893** | **0.007** | 0.365 | 0.300 | -0.310 | 0.417 | **0.857** | **0.014** | -0.071 | 0.879 | 0.750 | 0.052 |
|  | Serum eosinophils/mm^3^ | **0.903** | **0.000** | 0.400 | 0.286 | **0.810** | **0.015** | **-0.929** | **0.003** | 0.273 | 0.446 | -0.250 | 0.516 | **0.929** | **0.003** | 0.214 | 0.645 | **0.786** | **0.036** |
|  | Serum IgE IU/mL | 0.467 | 0.205 | 0.433 | 0.244 | 0.536 | 0.215 | -0.536 | 0.215 | 0.017 | 0.966 | -0.381 | 0.352 | 0.029 | 0.957 | 0.714 | 0.071 | 0.486 | 0.329 |

pAQLQ asthma quality of life questionnaire ACT: asthma control test; IgE: immunoglobulin E; FeNO: fractional exhaled nitric oxide measured at 50L/min.


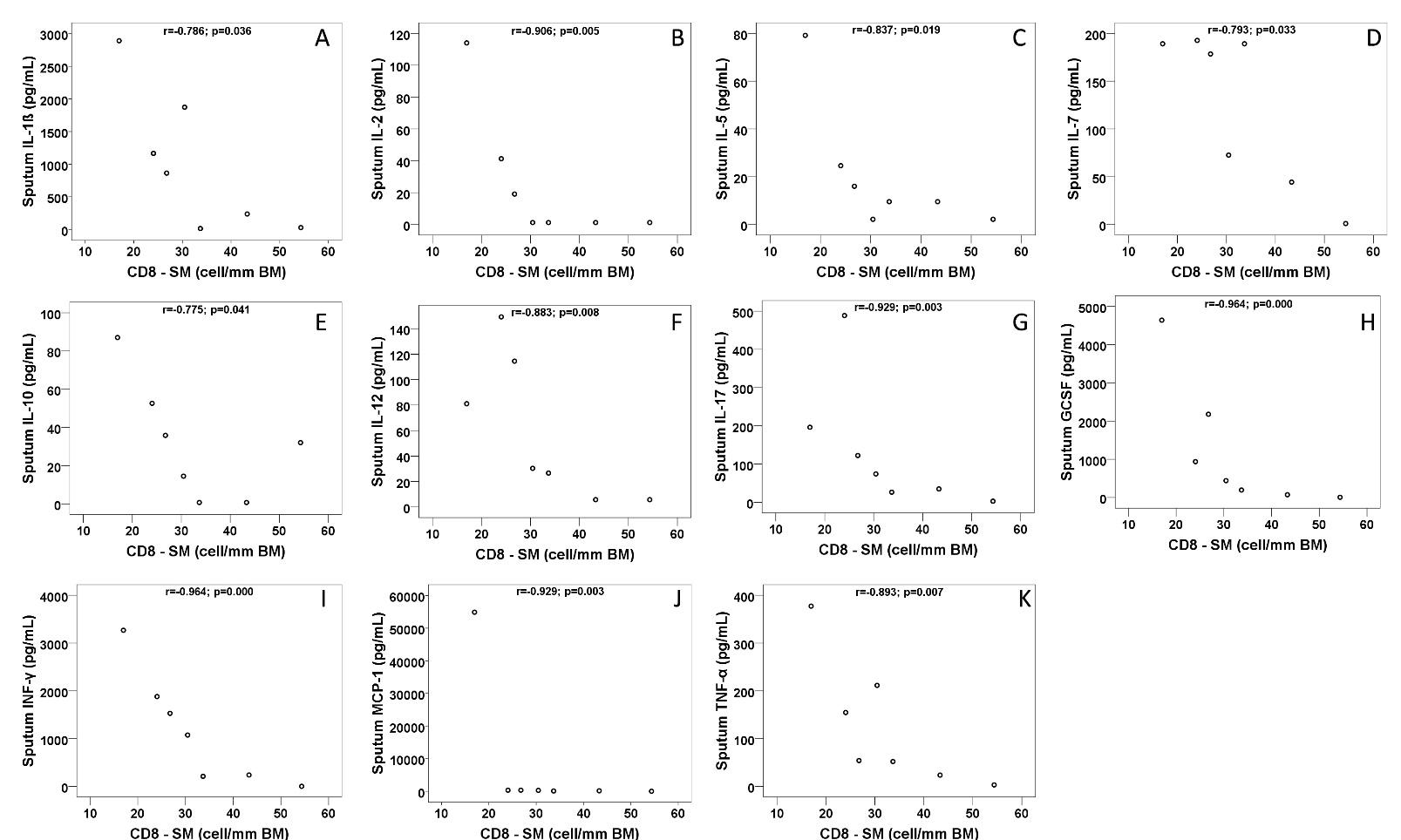


**Figure S2.** Correlation between CD8 T cells in bronchial biopsies and sputum interleukins in the patients with STRA (n=11)

**Figure S3**: Heatmap of correlation among cytokine levels in the sputum, bronchoalveolar lavage (BAL), and plasma. The correlation coefficients are color coded from deep red (−1) to deep blue (1).
